# Particle Swarm Optimization Algorithm-Based Design Method for Ultrasonic Transducers

**DOI:** 10.3390/mi11080715

**Published:** 2020-07-23

**Authors:** Dongdong Chen, Jianxin Zhao, Chunlong Fei, Di Li, Yuanbo Zhu, Zhaoxi Li, Rong Guo, Lifei Lou, Wei Feng, Yintang Yang

**Affiliations:** 1 School of Microelectronics, Xidian University, Xi’an 710071, China; ddchen@xidian.edu.cn (D.C.); jianxinzhao@stu.xidian.edu.cn (J.Z.); lidi2004@126.com (D.L.); xidianzhuyb@163.com (Y.Z.); lizhaoxivip@163.com (Z.L.); 18792732865@163.com (R.G.); ytyang@xidian.edu.cn (Y.Y.); 2 Shenzhen Institutes of Advanced Technology, Chinese Academy of Sciences, Shenzhen 518055, China; wei.feng@siat.ac.cn

**Keywords:** ultrasonic transducer, equivalent circuit model, optimization design, particle swarm optimization algorithm

## Abstract

In order to improve the fabrication efficiency and performance of an ultrasonic transducer (UT), a particle swarm optimization (PSO) algorithm-based design method was established and combined with an electrically equivalent circuit model. The relationship between the design and performance parameters of the UT is described by an electrically equivalent circuit model. Optimality criteria were established according to the desired performance; then, the design parameters were iteratively optimized using a PSO algorithm. The Pb(Zr_x_Ti_1−x_)O_3_ (PZT) ceramic UT was designed by the proposed method to verify its effectiveness. A center frequency of 6 MHz and a bandwidth of −6 dB (70%) were the desired performance characteristics. The optimized thicknesses of the piezoelectric and matching layers were 255 μm and 102 μm. The experimental results agree with those determined by the equivalent circuit model, and the center frequency and −6 dB bandwidth of the fabricated UT were 6.3 MHz and 68.25%, respectively, which verifies the effectiveness of the developed optimization design method.

## 1. Introduction

Pezoelectric ultrasonic transducers (UTs), as common energy conversion devices, have been widely used in nondestructive testing [1,2,3], ultrasonic positioning, [4,5] medical imaging and diagnoses [6,7,8]. The advantages of using such devices include their low cost and high efficiency, as well as the fact that they are safe, easy to use, and nonradiative. Typically, the performance indexes of UTs, such as center frequency (CF), −6 dB bandwidth (BW), sensitivity, etc. are mainly determined by their design parameters [9,10,11]. Therefore, it would be highly beneficial to establish an efficient design parameter optimization method for fabricating UTs with excellent performance.

In past decades, the design of UTs was mainly conducted by the equivalent circuit model (ECM) and finite element method (FEM). In 1970, the KLM (Krimholtz, Leedom and Mattaei) [12] model for UTs was proposed. In the KLM model, the acoustic and mechanical parameters of UTs are determined by the electrical components, which can accurately describe the effects of the design parameters on performance. Based on ECM, various kinds of ultrasonic devices with different center frequencies and broad bandwidths have been designed and fabricated. Based on the KLM model, Qian et al. [13] designed a high frequency (>30 MHz) UT using a new single-layer matching layer technology. Compared with double layer matching, the −6 dB BW of the fabricated UT with an acceptable loss in sensitivity is 70%. Also, Lau et al. [14] presented the multiple matching scheme for broadband phased-array transducers based on the KLM model. The designed UT with double matching layers achieved 110% of −6 dB BW and −46.5 dB of bidirectional insertion loss. In addition, Ma et al. [15] optimized the matching layer of a high frequency UT for vessel imaging based on the KLM model. The experimental results were basically consistent with the results determined by the KLM model, and the CF and −6 dB BW of the fabricated UT were 50.47 MHz and 74.94%, respectively. In recent years, some FEM software, including COMSOL Multiphysics, PZFlex, ANSYS and so on, has been used to design the UT. Bawiec et al. [16] developed a finite element model to optimize the geometric parameters of a 20–100 kHz flexural UT, which can be used for ultrasound-assisted chronic wound healing and percutaneous drug delivery. In addition, using PZFlex, Fei et al. [17] designed a (1−x)Pb(Mg_1/3_Nb_2/3_)O_3_−xPbTiO_3_ (PMN-PT) single crystal ultrasonic transducer with a half-concave geometry for intravenous ultrasound (IVUS) imaging. Compared with the flat ultrasonic transducer, the aforementioned ultrasonic transducer had higher CF and broader −6 dB BW. FEM can accurately simulate UTs with complex geometries and boundary conditions, and provides useful information for the design of UTs. Fiorillo et al. [18,19] designed a cochlear-shaped ultrasonic transducer similar to the biological cochlea, using the COMSOL Multiphysics software. The fabricated ultrasonic transducer had good emission and reception performance in the specific frequency range of 20–80 KHz. Using the COMSOL Multiphysics software, Chen et al. [20] designed a cone-shaped, ultrasonic transducer for three-dimensional ultrasonic positioning; it had a larger beam width and fewer receivers than commercial piezoelectric ceramic UT. In these traditional optimization design methods, the design parameters generally come from trial and error [21,22,23,24], which relies heavily on expert experience, thus greatly increasing the time and cost of the development cycle. Generally, the FEM method has a heavy calculation burden, and is highly time-consuming, which decreases the efficiency of the optimization design for UTs. In contrast, the ECM is easy to use, and has a low computation burden. So, it can be easily combined with an optimization algorithm to develop an effective design method for UTs. 

Due to the complex effects of design parameters on the performance of UTs, the optimization of design parameters is the primary consideration. Generally, traditional optimization methods, such as the Newton, quasi-Newton and conjugate direction methods, obtain the optimal solution using operations of derivatives [25,26,27] which require the objective function be derivable. However, this condition cannot be satisfied in the optimization design of UTs. In recent years, intelligent optimization algorithms, including the genetic algorithm, particle swarm optimization (PSO) algorithm, the ant colony algorithm and the simulated annealing algorithm have been proposed and widely used in the processing optimization of metals and alloys [28,29] and the optimization design of pressure sensors and gyroscopes [30,31,32], as the objective function need not be derivable. Therefore, the intelligent optimization algorithm can be utilized in the optimization design of UTs.

In this paper, an intelligent, optimization algorithm-based design method for UTs is established, combined with the ECM. In the proposed method, the relationship between the design and performance parameters of UTs is described by ECM. The optimality criteria were established based on the performance parameters, and the PSO algorithm was used to optimize design parameters based on the established optimality criteria. The UT was fabricated using the optimized design parameters, and its performance was tested to verify the effectiveness of the proposed method.

## 2. Optimization Design Method for Ultrasonic Transducer

**Figure 1:**
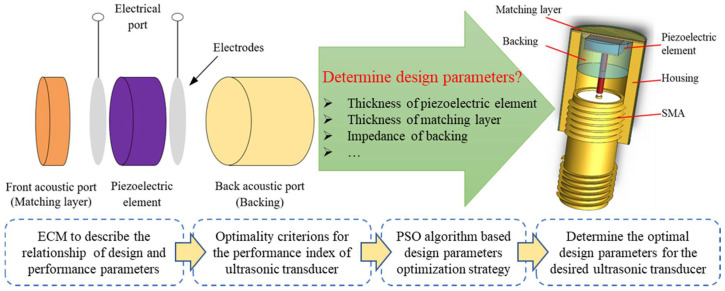
Schematic diagram of the developed design optimization method for an ultrasonic transducer.

Generally, the performance of a UT is affected by its design parameters once the functional materials (typically, piezoelectric material, matching and backing materials) have been selected. Therefore, the optimization of design parameters plays an important role in fabricating the desired UT. In this work, a design optimization method for UTs is proposed. The ECM was adopted to describe the relationship between the design and performance parameters. According to the optimality criteria for the performance indexes of UTs, the PSO algorithm was adopted to optimize the design parameters of the UT. Figure 1 shows a flowchart of the process.

### 2.1. Equivalent Circuit Model

**Figure 2:**
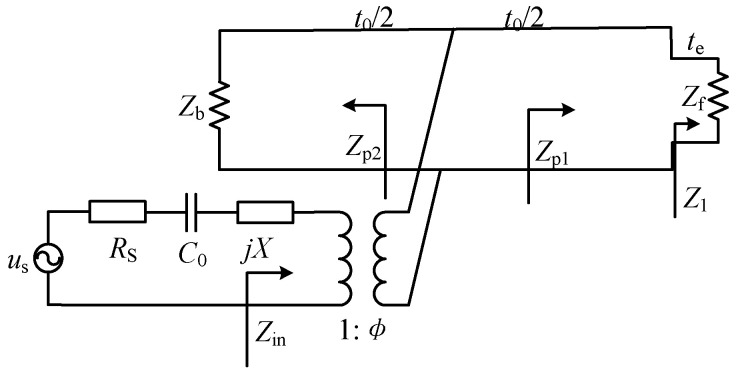
Equivalent circuit for the thickness mode UT with one matching layer.

The equivalent circuit for the thickness mode UT is shown in Figure 2. It should be noted that the electrodes are sufficiently thin so that their influence on wave propagation through the piezoelectric medium can be ignored. The ECM allows an intuitive approach to be used in the design of the UT, and the two acoustic ports representing the front and back face of the UT, respectively. In this model, the piezoelectric UT is described by an acoustic transmission line tapped at its center, and driven by a perfect transformer of ratio (1:Φ). In addition, the effects of matching and backing layers on the UT can be readily included.

In the equivalent circuit for the thickness-expander disc (as shown in Figure 2), the element values can be calculated as [33]

(1) $$\left\{ \begin{array}{l} C_{0}=\varepsilon_{33}^{S}\frac{S}{t_{0}}\\ \omega_{0}=\pi \frac{c}{t_{0}}\\ Z_{0}=\rho cS\\ \frac{1}{\Phi }=k_{t}\sqrt{\frac{\pi }{\omega_{0}C_{0}Z_{0}}}\sin c\left(\frac{\omega }{\omega_{0}}\right)\\ X=k_{t}^{2}\frac{1}{\omega C_{0}}\sin c\left(\frac{\omega }{\omega_{0}}\right) \end{array}\right.$$

The parameters are defined as follows: $C_{0}$ piezoceramic clamped capacity; $t_{0}$ thickness of the piezoelectric element; S area of the piezoelectric element; $\varepsilon_{33}^{S}$ ceramic permittivity with zero or constant strain; $\omega_{0}$ half-wavelength resonant frequency; c longitudinal velocity of the piezoelectric element; $Z_{0}$ acoustic impedance of the piezoelectric layer; ρ density of the piezoelectric element; Φ ratio of transformer; $k_{t}$ effective piezoelectric coupling coefficient of piezoelectric element; ω angular frequency; X reactance of piezoelectric element.

Based on the transmission line theory, the input impedance can be deduced by straightforward circuit analysis, and can be expressed as

(2) $$Z_{in}=\frac{1}{j\omega C_{0}}+jX+\frac{1}{\Phi^{2}}\cdot \frac{Z_{p1}Z_{p2}}{Z_{p1}+Z_{p2}}$$

where $Z_{p1}$ and $Z_{p2}$ are the input impedance of the acoustic transmission line looking towards the front and back acoustic ports, respectively, and they can be expressed as [10]

(3) {Zp1=Z0Z1cos(k0t02)+jZ0sin(k0t02)Z0cos(k0t02)+jZ1sin(k0t02)Zp2=Z0Zbcos(k0t02)+jZ0sin(k0t02)Z0cos(k0t02)+jZbsin(k0t02)

where $k_{0}$ is wave number of the piezoelectric element, and $Z_{b}$ is the acoustic impedance of the backing. $Z_{1}$ can be calculated by

(4) $$Z_{1}=Z_{e}\frac{Z_{f}\cos\left(k_{e}t_{e}\right)+jZ_{e}\sin\left(k_{e}t_{e}\right)}{Z_{e}\cos\left(k_{e}t_{e}\right)+jZ_{f}\sin\left(k_{e}t_{e}\right)}$$

where $k_{e}$ and $t_{e}$ are the wave number and thickness of matching layer, respectively. $Z_{e}$ and $Z_{f}$ are the acoustic impedances of the matching layer and front load, respectively.

According to the theory of the acoustic transmission line, the transmission matrix of a piezoelectric layer with half-thickness $t_{0}/2$ can be expressed as [34,35]

(5) N0=[cos(k0t02)jZ0sin(k0t02)jsin(k0t02)Z0cos(k0t02)]

Similarly, the transmission matrix of the matching layer with thickness $t_{e}$ can be expressed as

(6) N1=[cos(kete)jZesin(kete)jsin(kete)Zecos(kete)]

The UT is a multilayer structure, and the matching layer is added at the front of the piezoelectric layer. Therefore, the piezoelectric layer and matching layer can be regarded as two acoustic transmission lines. The total transmission matrix of the output can be obtained by multiplying each transmission matrix, and can be expressed as

(7) $$N=N_{0}\cdot N_{1}=\left[\begin{array}{cc} N_{11} & N_{12}\\ N_{21} & N_{22} \end{array}\right]$$

Based on the above analysis, the output voltage of the UT can be calculated as

(8) $$u_{f}=\frac{u_{s}}{\Phi }\cdot \frac{Z_{p2}Z_{f}}{\left(R_{s}+Z_{in}\right)\left(Z_{p1}+Z_{p2}\right)}\cdot \frac{1}{N_{21}Z_{f}+N_{22}}\left(R_{s}=50\Omega \right)$$

By using the Fourier transform method, the pulse/echo response of the UT can be predicted with good accuracy. Then, the design of the UT can be conducted based on ECM. In this study, the design parameters of the UT are the thicknesses of the piezoelectric and matching layers, and the performance parameters include center frequency (CF) and bandwidth (BW), which can be calculated as [36]

(9) $$CF=\frac{f_{1}+f_{2}}{2}$$

(10) $$BW=\frac{f_{2}-f_{1}}{CF}\times 100\%$$

where $f_{1}$ and $f_{2}$ represent the lower and upper frequencies at which the amplitude drops to the −6 dB peak. 

### 2.2. Optimality Criteria of Ultrasonic Transducer 

The optimality criteria of the UT are established based on the performance parameters, and can be expressed as

(11) $$J=\alpha \left(CF-CF_{\mathrm{des}}\right)+\beta \left(BW-BW_{\mathrm{des}}\right)$$

where $CF_{\mathrm{des}}$ and $BW_{\mathrm{des}}$ represent the desired CF and BW, respectively, and α and β are their weight coefficients.

The optimality criteria should be normalized to avoid the effects of magnitude; the normalized optimality criteria can be calculated as

(12) $$J=\alpha \left(\frac{CF-CF_{\mathrm{des}}}{CF_{\max}-CF_{\min}}\right)+\beta \left(\frac{BW-BW_{\mathrm{des}}}{BW_{\max}-BW_{\min}}\right)$$

where $CF_{\min}$, $CF_{\max}$, $BW_{\min}$ and $BW_{\max}$ are the minimum and maximum values of CF and BW, respectively.

### 2.3. Design Parameter Optimization Method under the Framework of PSO Algorithm

**Figure 3:**
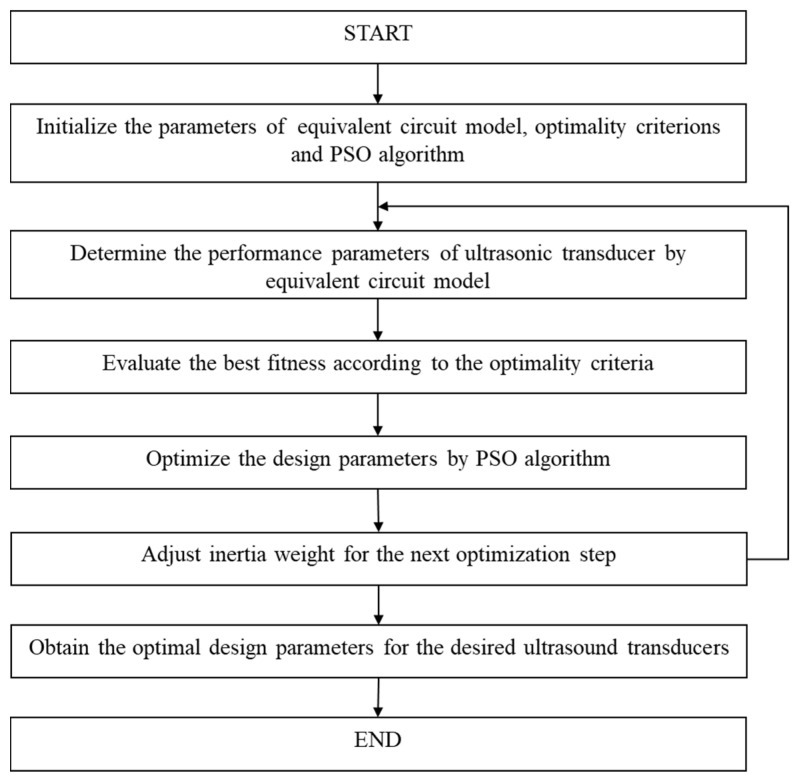
Flowchart of design parameter optimization method within the framework of the PSO algorithm.

Based on the optimality criteria and ECM, a design parameter optimization method is proposed under the framework of the PSO algorithm. Because the PSO algorithm with linear decreasing inertia weight (PSO-LDIW) has superior search abilities [37,38], it was adopted to optimize the design parameters of the UT. A flowchart of the proposed design parameter optimization method is shown in Figure 3; it comprises six main steps:

Step 1: Initialize the parameters of ECM, optimality criteria and the PSO algorithm. The parameters of ECM, optimality criteria and the PSO algorithm should be presented according to the specific UT.

Step 2: Determine the performance parameters of the UT by ECM. Based on the presented design parameters, the performance of the UT can be determined by ECM.

Step 3: Evaluate the best fitness according to the optimality criteria. Based on the performance parameters determined by ECM, the best fitness can be calculated by the established optimality criteria.

Step 4: Determine the optimal design parameters by using the PSO algorithm. According to the optimality criteria, the design parameters can be optimized by the PSO algorithm. The optimal solution can be calculated as

(13) $$v_{i}(t+1)=w\left(iter\right)v_{i}(t)+c_{1}r_{1}\left(p_{i}-x_{i}(t)\right)+c_{2}r_{2}\left(p_{g}-x_{i}(t)\right)$$

(14) $$x_{i}(t+1)=x_{i}(t)+v_{i}(t+1)$$

Step 5: Adjust the inertia weight for the next optimization step. In the PSO-LDIW algorithm, the inertia weight is linearly decreased for the next optimization step, and can be adjusted as

(15) $$w(iter)=\frac{iter_{\max}-iter}{iter_{\max}}\left(w_{\max}-w_{\min}\right)+w_{\min}$$

Step 6: Obtain the optimal design parameters for the desired ultrasound transducers.

## 3. Application and Verification of the Developed Optimization Design Method

In this work, a 6 MHz UT was designed and fabricated to verify the effectiveness of the proposed optimization design method.

### 3.1. Optimization Design of Ultrasonic Transducer

The designed UT includes three functional layers: the piezoelectric layer, the backing layer and the matching layer; its ECM is presented in Section 2.1. Table 1 shows the parameters for ECM. It should be noted that the design parameters are the thicknesses of the piezoelectric and matching layers; the backing layer is determined ideally.

The parameters of optimality criteria and the PSO-LDIW algorithm for the desired UT are shown in Table 2. The desired CF and BW are 6 MHz and 70%, respectively. To fabricate a UT with high BW, the weight coefficient of BW must be 0.6, which is larger than that of CF. In the PSO-LDIW algorithm, the inertia weight is decreased from 0.9 to 0.4.

**Figure 4:**
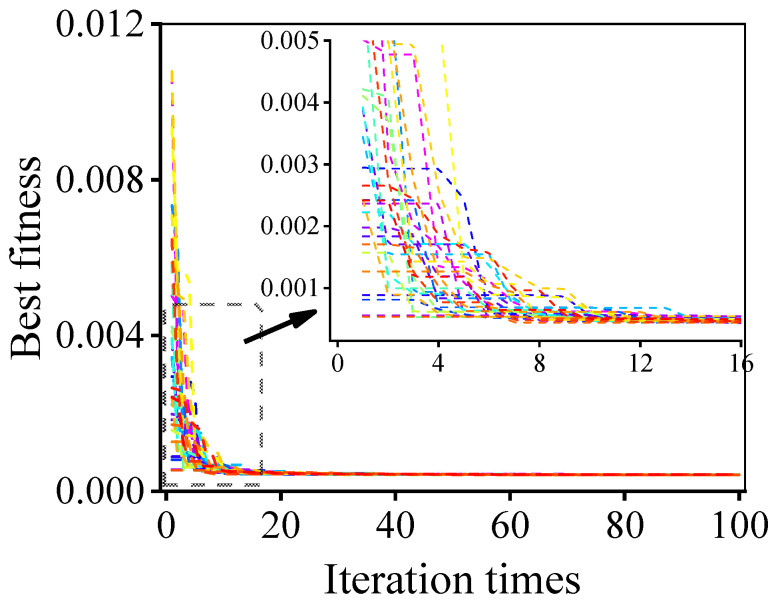
Best fitness of the developed optimization design method for ultrasonic transducer.

In this research, the optimization design method for the UT was determined by 30 independent runs to decrease the effect of random error in the PSO-LDIW algorithm. Figure 4 shows the best fitness of the proposed method. In order to ensure the accuracy and efficiency of the simulation, the number of iterations was set to 100. The final best fitness approached zero, which implied that the desired performance of the UT had been achieved. Also, the similar best fitness of 30 independent runs indicated that the proposed method is stable and effective.

**Figure 5:**
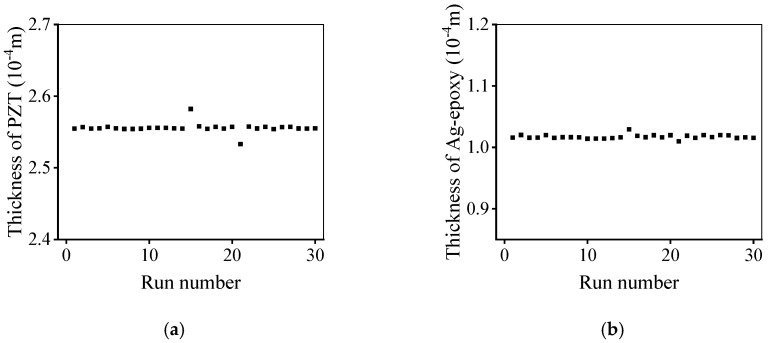
Optimized design parameters of ultrasonic transducer: (**a**) thickness of piezoelectric layer; (**b**) thickness of matching layer.

**Figure 6:**
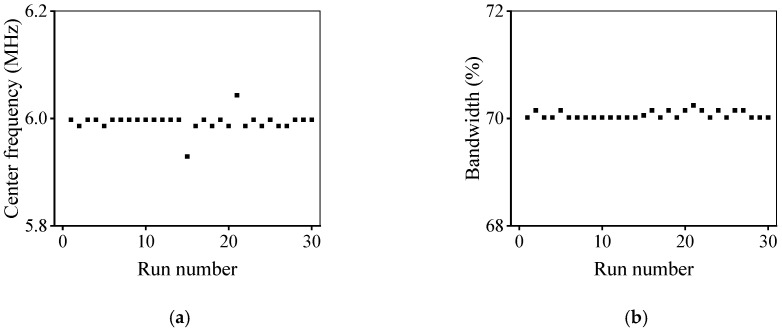
Performance parameters of ultrasonic transducer obtained at the optimized design parameters: (**a**) center frequency; (**b**) −6 dB bandwidth.

Figure 5 shows the design parameters of the UT optimized by the developed method. After 30 independent runs, the optimized design parameters were relatively consistent, and the thicknesses of piezoelectric and matching layers were around 255 μm and 102 μm, respectively. The performance parameters of the optimized UT are shown in Figure 6, and the CF and −6 dB BW were about 6 MHz and 70%, respectively, i.e., almost achieving the desired performance. The design parameters of the UT can be effectively optimized by the developed optimization design method.

**Figure 7:**
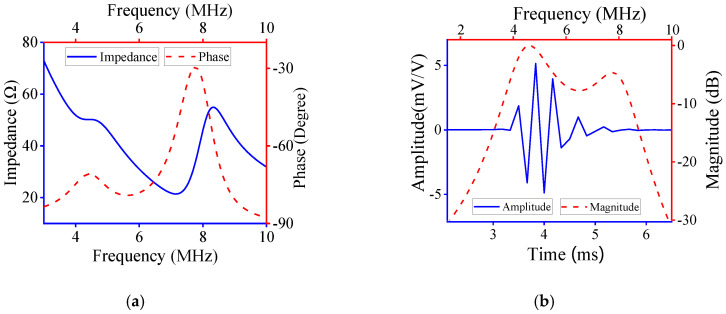
Equivalent circuit model results: (**a**) electrical impedance and phase; (**b**) time-domain pulse/echo response and normalized frequency spectrum.

Based on the design parameters determined by the developed method, the ECM results are shown in in Figure 7. Obviously, the electrical impedance and phase vary with frequency, and there were two peaks, as shown in Figure 7a. Figure 7b shows the time-domain pulse/echo response and normalized frequency spectrum, and the CF and −6 dB BW are 5.99 MHz and 70.40%, which agree with the desired performance characteristics.

### 3.2. Verification

**Figure 8:**
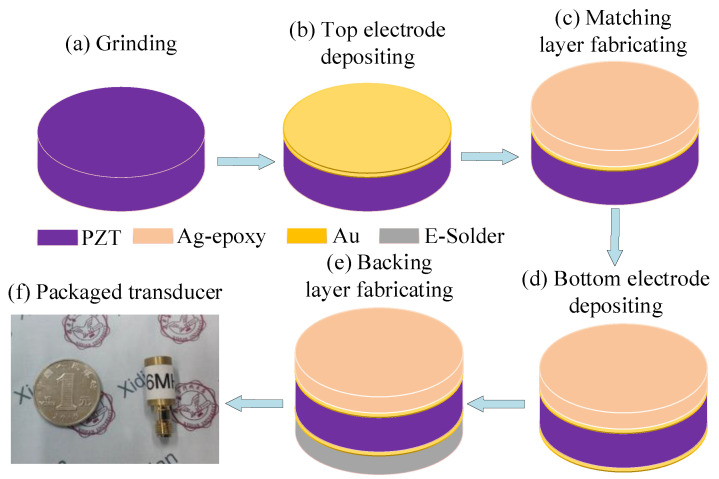
Schematic of the fabrication process of ultrasonic transducer.

In order to validate the effectiveness of the proposed optimization design method, a UT was fabricated according to the optimized design parameters. The fabrication process of the UT is shown in Figure 8. Firstly, the prepared piezoelectric disc was ground to the desired thickness (255 μm). Then, the top Au electrode with a thickness of 20 nm was patterned and deposited on the PZT (PIC255, Physik Instrumente, Karlsruhe/Palmbach, Germany) layer by the sputtering technique. Subsequently, Ag-epoxy with a ratio of 1.25:3 was used as the matching layer to broaden the bandwidth of the UT, and then it was ground to a thickness of about 102 μm after curing for 24 h at 300 K. Then, the electrode was plated on the back of the piezoelectric disc. An E-Solder 3022 with a thickness of 1.5 cm was used as backing layer to suppress unnecessary vibrations and eliminate the reflection on the back of the piezoelectric wafer. Finally, the UT was packaged. The fabricated UT is shown in Figure 8f.

**Figure 9:**
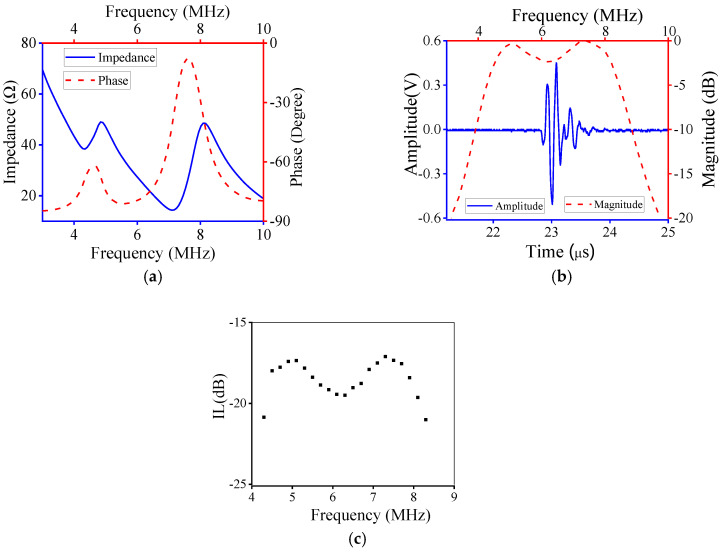
Test results of the fabricated ultrasonic transducer: (**a**) electrical impedance and phase; (**b**) time-domain pulse/echo response and normalized frequency spectrum; (**c**) insertion loss.

The fabricated UT was tested to verify its performance; the detailed testing procedure is described in our previous research [39]. The test results of the fabricated UT are shown in Figure 9. Obviously, the electrical impedance and phase of experimental results were almost consistent with those determined by ECM. Also, the time-domain pulse/echo response and normalized frequency spectrum of experimental results agreed with those simulated by ECM. Figure 9c shows the two-way insertion loss (IL) of the fabricated UT. It can be seen that the maximum IL was about −17.2 dB at 5 MHz and 7.3 MHz, which indicates that the fabricated transducer has good sensitivity. The experimental CF and −6 dB BW were 6.3 MHz and 68.25%, respectively, i.e., nearly achieving the desired performance. Therefore, the proposed design method is practicable and efficient.

### 3.3. Comparison and Discussion

The performance parameters of the designed, simulated and fabricated UT are presented in Table 3. The CF and −6 dB BW determined by ECM agreed with the designed ones. The CF of the fabricated UT was 6.30 MHz, which was slightly higher than that of the designed CF. However, the −6 dB BW of the fabricated UT was 68.25%, i.e., slightly lower than the designed BW. The resonance at 4 MHz, as shown in Figure 7a and Figure 9a, was caused by the matching layer; the latter was more evident than the former. Due to the deviation of the fabrication process for the UT, the testing performance varied within a certain range, and the relative error of the testing result was less than 10%. The relative errors of CF and −6 dB BW were 5% and 2.5%, respectively. Therefore, the established optimization method could be used to design UTs with excellent performance.

As shown in Table 3, the ECM can accurately describe the performance of the UT. In addition, the PSO algorithm is an effective optimization algorithm and can overcome the limitations of traditional optimization algorithms. Therefore, the optimization results are reasonable and effective. Furthermore, the developed optimization method can be automatically conducted on a computer, and does not rely on human intervention, which effectively decreases the time and cost of the development. In this study, only the fundamental performance characteristics (CF and −6 dB BW) were considered. In future research, the performance characteristics such as resolution, sensitivity and energy conversion efficiency, etc. will be comprehensively taken into consideration for the design and fabrication of a high-performance UT.

## 4. Conclusions

A PSO algorithm-based design method for UTs was developed combined with the ECM. In the developed method, the ECM was utilized to describe the effects of design parameters on the performance of the UT. The performance parameters, including CF and −6 dB BW, were considered to establish the optimality criteria of the UT. Based on ECM, the PSO algorithm was utilized to iteratively optimize the design parameters of the UT according to the established optimality criteria. The optimized thicknesses of the piezoelectric and matching layers were 255 μm and 102 μm, respectively. The CF and −6 dB BW determined by ECM were 5.99 MHz and 70.40%, respectively. In addition, the CF and −6 dB BW of the fabricated UT were 6.3 MHz and 68.25%, respectively, almost achieving the desired performance. Therefore, the design parameters can be effectively optimized by the proposed optimization method to fabricate the desired UT.

## Figures and Tables

**Table 1:** Parameters for equivalent circuit model.

Materials	Function	Velocity (m/s)	Density (kg/m^3^)	Acoustic Impedance (MRayl)	Dielectric Constant
PZT (PIC255)	Piezoelectric layer	4044	7860	31.79	748
Gold	Electrode	3240	19,700	63.8	-
Water	Front load	1540	1000	1.54	-
E-Solder	Backing layer	1850	3200	5.92	-
Ag-epoxy	Matching layer	1900	3100	5.89	-

**Table 2:** Parameters of optimality criteria and PSO-LDIW algorithm.

**Parameters of Optimality Criteria**	Desired CF	$CF_{\mathrm{des}}=6\;\mathrm{MHz}$
	Desired BW	$BW_{\mathrm{des}}=70\%$
	Weight Coefficients	α=0.4, β=0.6
**Parameters of PSO-LDIW algorithm**	Constant parameters	$c_{1}=2$, $c_{2}=2$
	Range of inertia weight	w∈[0.4,0.9]
	Maximum generation	MaxG=100
	Population size	N= 50
	Range of particle position	$x_{t_{0}}\in [2,10]\times 10^{-4}$, $x_{t_{e}}\in [1,50]\times 10^{-5}$
	Range of particle velocity	$v_{t_{0}}\in [-5,5]\times 10^{-4}$, $v_{t_{e}}\in [-5,5]\times 10^{-5}$

**Table 3:** Performance of designed, simulated and fabricated transducers.

Performance	Pulse-Echo			
	*f*_1_(MHz)	*f*_2_(MHz)	*CF*(MHz)	*BW*(%)
Design	/	/	6	70.00
Model	3.89	8.11	5.99	70.40
Experiment	4.15	8.45	6.30	68.25

## Symbols

$t_{0}$	Thickness of the piezoelectric material
$t_{e}$	Thickness of the matching layer
$C_{0}$	Piezoceramic clamped capacity
S	Area of the transducer
$\varepsilon_{33}^{S}$	Ceramic permittivity with zero or constant strain
$Z_{0}$	Acoustic impedance of the piezoelectric layer
ρ	Density of the piezoelectric material
c	Longitudinal velocity of the piezoelectric material
Φ	Ratio of transformer
$k_{t}$	Effective piezoelectric coupling coefficient
$\omega_{0}$	Resonant frequency
X	Reactance of piezoelectric element
$Z_{p1}$	Input impedance of the acoustic transmission line looking towards the front acoustic port
$Z_{p2}$	Input impedance of the acoustic transmission line looking towards the back acoustic port
$k_{0}$	Wave number of the piezoelectric material
$k_{e}$	Wave number of the matching layer
$Z_{e}$	Acoustic impedance of the matching layer
$Z_{f}$	Acoustic impedance of front load(acoustic impedance of water)
$Z_{b}$	Acoustic impedance of the backing
CF	Center frequency
BW	Bandwidth
$f_{1}$	Lower frequency at which the amplitude drops to the −6 dB peak
$f_{2}$	Upper frequency at which the amplitude drops to the −6 dB peak
J	Optimality criteria of ultrasonic transducer
$CF_{\mathrm{des}}$	Desired *CF*
$BW_{\mathrm{des}}$	Desired *BW*
α	Weight coefficients of *CF*_des_
β	Weight coefficients of *BW*_des_
$CF_{\min}$	Minimum values of *CF*
$CF_{\max}$	Maximum values of *CF*
$BW_{\min}$	Minimum values of *BW*
$BW_{\max}$	Maximum values of *BW*
$v_{i}$	The *i*th particle’s velocity and position
$x_{i}$	The *i*th particle’s position
w	Inertia weight
$c_{1}$, $c_{2}$	Two constants
$p_{i}$	Best previous positions of the *i*th individual in current generation
$p_{g}$	Best previous positions of the *i*th all particles in current generation
$r_{1}$, $r_{2}$	Two random values distributed in the range of [0, 1]
iter	Current iteration
$iter_{\max}$	Maximum of the current iteration
$w_{\max}$	Maximum of inertia weight
$w_{\min}$	Minimum of inertia weight
